# Supplementary material for: miRScore: A rapid and precise microRNA validation tool
Source: PLoS Comput Biol. 2025 Nov 3;21(11):e1013663. doi: 10.1371/journal.pcbi.1013663 (PMC12594335; doi:10.1371/journal.pcbi.1013663)
Supplement: S5 File — (PDF) [file pcbi.1013663.s005.pdf]

# Pipeline for reproducing annotation results from Vanek et al., 2025

### Reference genomes used for analysis:

Arabidopsis thaliana genome: GCF_000001735.4_TAIR10.1_genomic.fa
Zea mays genome: Zm-B97-REFERENCE-NAM-5.0.fa
Oryza sative genome: osa1_r7.asm.fa
Striga hermonthica genome: GCA_902706635.1_SHERM_genomic.fa

Arabidopsis thaliana FASTQ: SRR218096, SRR218092, SRR218099, SRR218098, SRR218085
Zea mays FASTQ: SRR032087, SRR023088, SRR032089, SRR032090, SRR032091
Oryza sative FASTQ: SRR037234, SRR037235, SRR037236, SRR037237, SRR218085
Striga hermonthica FASTQ: GEO accession GSE282265

All example commands are for Arabidopsis thaliana, but similar commands were used for each species.

## miRador

miRador was run in accordance with their GitHub page: https://github.com/rkweku/miRador.
The 'miRador.ini' file is provided in supplemental data. sRNA-seq data was trimmed prior to running miRador using ShortStack trim feature.

Upon running miRScore, several precursors were found not to have mature miRNAs in the finalAnnotatedCandidates.fa and were removed.

### miRador output for miRScore input:

**miRNAs:** finalAnnotatedCandidates.fa
**precursors:** precursors.fa
**FASTQ files:** trimmed FASTQ files input to miRador

## ShortStack

ShortStack was run using the following command:

```
ShortStack --genomefile GCF_000001735.4_TAIR10.1_genomic.fa --outdir
ss_ath --autotrim --threads 5 --dn_mirna --readfile fastq/*
```

The following AWK commands were used to parse the mature miRNA and precursor sequences from the miRDP2 output:

```
#mature miRNAs
    cat mir.fasta| grep -A1 'mature\|star' | grep -v -e '--'> mirnas.fa
```

```
#Adjust names to be more concise
cat mirnas.fa| sed 's/::.*//' >mirnas_adj.fa

#hairpins
cat mir.fasta | grep -v "mature\|star" | grep -A1 ">" | grep -v -e '--
'>hairpins.fa

#Adjust names to be more concise
cat hairpins.fa| sed 's/::.*//' >hairpin_adj.fa
```

### ShortStack output for miRScore input:

**miRNAs:** mirnas_adj.fa
**precursors:** hairpin_adj.fa
**FASTQ files:** FASTQ files input to ShortStack

## mirdeep-p2

miRDeep-P2 was run using several commands in accordance with the developer's pipeline: https://github.com/TF-Chan-Lab/miRDeep-P2_pipeline

sRNA-seq data was trimmed prior. These are the same files used for miRador analysis.

1. Reference genome index file was created:

```
bowtie-build GCF_000001735.4_TAIR10.1_genomic.fa athGenome
```

2. Run miRDeep-P2 miRNA prediction:

```
mkdir result
miRDP2-v1.1.4_pipeline.bash -g GCF_000001735.4_TAIR10.1_genomic.fa -x
athGenome -q -b fastq_list.txt -o result
```

Note that the 'fastq_list.txt' is a user-generated list of your FASTQ files. Also note that at this step, an error regarding the .ori files appeared and required moving the files back a directory.

3. Extracted predicted miRNA sequences from miRDP2 results:

```
perl ./miRDeep-P2_pipeline-main/script/parse_miRDP2_prediction.pl
miRDP2_predictions_list.txt miRDP2
```

Note that the 'miRDP2_predictions_list.txt' is a user-generated file containing the locations of all prediction files generated from step 2.

The following AWK commands were used to parse the mature miRNA and precursor sequences from the miRDP2 output:

```
#mature miRNAs
awk '/^>/ {id=$1; next} {split($0, a, ","); print id "\n" a[length(a)]}'
miRDP2_mature.fa > mature.fa

#hairpins
python prec_prim.py
```

prec_prim.py is a custom python script included in supplemental used to extract hairpins from mature miRNAs which had the precursor in the header.

### miRDeep-P2 output for miRScore input:

**miRNAs:** mature.fa
**precursors:** hairpin.fa
**FASTQ files:** trimmed FASTQ files input to miRDeep
