# Supplementary material for: miRScore: A rapid and precise microRNA validation tool
Source: PLoS Comput Biol. 2025 Nov 3;21(11):e1013663. doi: 10.1371/journal.pcbi.1013663 (PMC12594335; doi:10.1371/journal.pcbi.1013663)
Supplement: S6 File — (PDF) [file pcbi.1013663.s006.pdf]

Preparing miRBase and MirGeneDB data for miRScore

miRBase

Example : *Arabidopsis thaliana*

1. Download the 'mature.fa' and 'hairpin.fa' file from miRBase download page.

```
#precursor sequences
wget -O hairpin.fa "https://mirbase.org/download/hairpin.fa"

#mature sequences
wget -O mature.fa "https://mirbase.org/download/mature.fa"
```

2. Prepare hairpin file by converting to single line FASTA

```
awk '/^>/ {if (seq) print seq; print; seq=""; next} {seq = seq $0} END
{print seq}' hairpin.fa > singleline_hairpin.fa
```

3. Parse out ath entries and adjust names to be more concise. The mature miRNA and hairpin names must match!

```
# Hairpin sequences
cat singleline_hairpin.fa| grep -A1 "thaliana" |grep -v -e '--' >
ath_hairpins.fa

sed -i 's/ .*//' ath_hairpins.fa

# miRNA and miRNA* sequences
cat mature.fa| grep -A1 "thaliana"|grep -v -e '--'> ath_mature.fa

sed -i 's/ .*//' ath_mature.fa
```

4. Download fastq files

```
mkdir fastq
cd fastq
fasterq-dump SRR218096
```

At this stage, if you run miRScore it will inform you of the following:

Error! The following entries in the MIRNA hairpin file 'ath_hp.fa' have no mature sequences that match their identifiers in file 'ath_mat.fa'. ['ath-MIR161', 'ath-MIR779', 'ath-MIR780', 'ath-MIR869', 'ath-MIR1886'] Please check all hairpins in the hairpin FASTA file have miRNAs in the mature FASTA file with the same name. If you have multiple miRNAs assigned to a single locus (i.e. osa-miR159a.1/osa-miR159a.2 to osa-MIR159a) please run 'hairpinHelper' then rerun miRScore with the'miRScore_adjusted_hairpins.fa' file as the hairpin FASTA input.

So you need to run hairpinHelper like so:

```
hairpinHelper -mature ath_mature.fa -hairpin ath_hairpins.fa
```

Alternatively, you could just remove those entries from the hairpin file.

5. Run miRScore using adjusted hairpins

```
miRScore -mature ath_mature.fa -hairpin miRScore_adjusted_hairpins.fa -
fastq fastq/* -autotrim -kingdom plant -out ath_results
```

MirGeneDB

Example : *Homo sapiens*

1. Download precursors w/ flank and mature sequences

```
#precursors sequences
wget -O hsa-pri.fa "https://mirgenedb.org/fasta/hsa?pri=1"

#mature sequences
wget -O hsa-mat.fa "https://mirgenedb.org/fasta/hsa?mat=1"
```

2. Adjust precursors to not have '_pri' and miRNAs need to have '-3p' not "_3p" in order for miRScore to recognize.

```
#Precursor
sed -i 's/_pri//' hsa-pri.fa

#Mature
sed -i '/^>/ s/_\([35]p\)/-\1/' hsa-mat.fa
```

3. Download fastq files

```
mkdir fastq
cd fastq
fasterq-dump SRR518956
```

4. Run miRScore

```
miRScore -mature hsa-mat.fa -hairpin hsa-pri.fa -kingdom animal -fastq fastq/* -autotrim -out hsa_results
```
