# Supplementary material for: miRScore: A rapid and precise microRNA validation tool
Source: PLoS Comput Biol. 2025 Nov 3;21(11):e1013663. doi: 10.1371/journal.pcbi.1013663 (PMC12594335; doi:10.1371/journal.pcbi.1013663)
Supplement: S1 Fig — (A) When counting miRNA duplex reads, a variance window of -/ + 1 nt from the indexed start/stop position of the miRNA and miRNA*. Reads which start and stop within this window are counted towards the total miRNA duplex count and used to determine precision. (B) Example of reads which are included in total count of hsa-mir-212 miRNA (red), miRNA* (blue), and those that are not included in count (black). Read length (len) and number of reads aligned at that position (al) can be found on the right side. (C) Example of reads included in ath-MIR167a miRNA (red), miRNA* (blue), and those that are not included (black). (DOCX) [file pcbi.1013663.s008.docx]

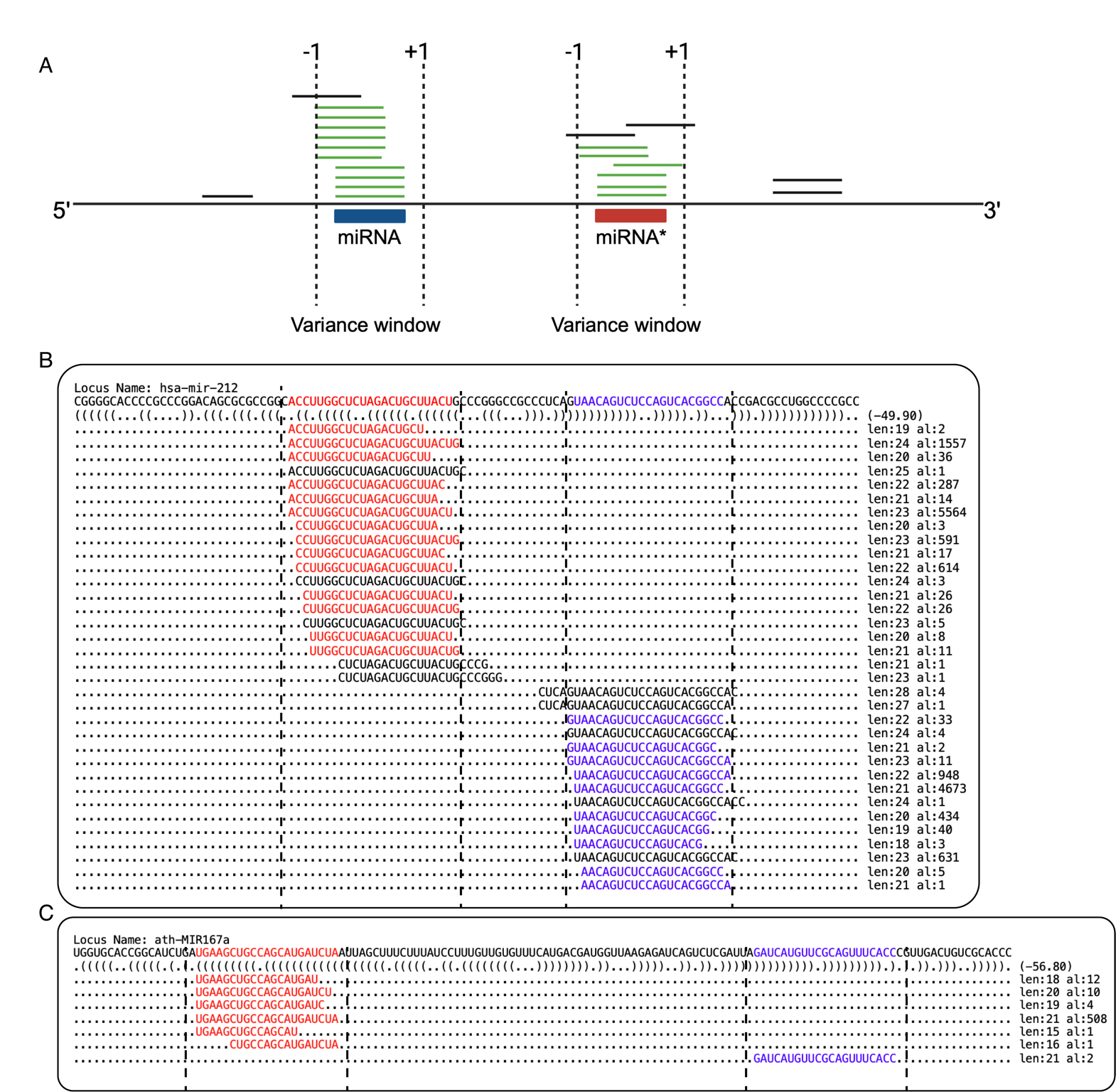


**Supplemental Figure S1.** Explanation of read alignment, precision, and variance. (A) When counting miRNA duplex reads, a variance window of -/+ 1 nt from the indexed start/stop position of the miRNA and miRNA*. Reads which start and stop within this window are counted towards the total miRNA duplex count and used to determine precision. (B) Example of reads which are included in total count of has-mir-212 miRNA (red), miRNA* (blue), and those that are not included in count (black). Read length (len) and number of reads aligned at that position (al) can be found on the right side. (C) Example of reads included in ath-MIR167a miRNA (red), miRNA* (blue), and those that are not included (black).
