# Supplementary material for: miRScore: A rapid and precise microRNA validation tool
Source: PLoS Comput Biol. 2025 Nov 3;21(11):e1013663. doi: 10.1371/journal.pcbi.1013663 (PMC12594335; doi:10.1371/journal.pcbi.1013663)
Supplement: S2 Fig — See S2 File for source data. (A) Mus musculus (mmu) mirbase MIRNA results and flags. (B) Homo sapiens (hsa) mirbase MIRNA results and flags. (C) mmu MirGeneDB MIRNA results and flags. (D) hsa MirGeneDB MIRNA results and flags. (DOCX) [file pcbi.1013663.s009.docx]

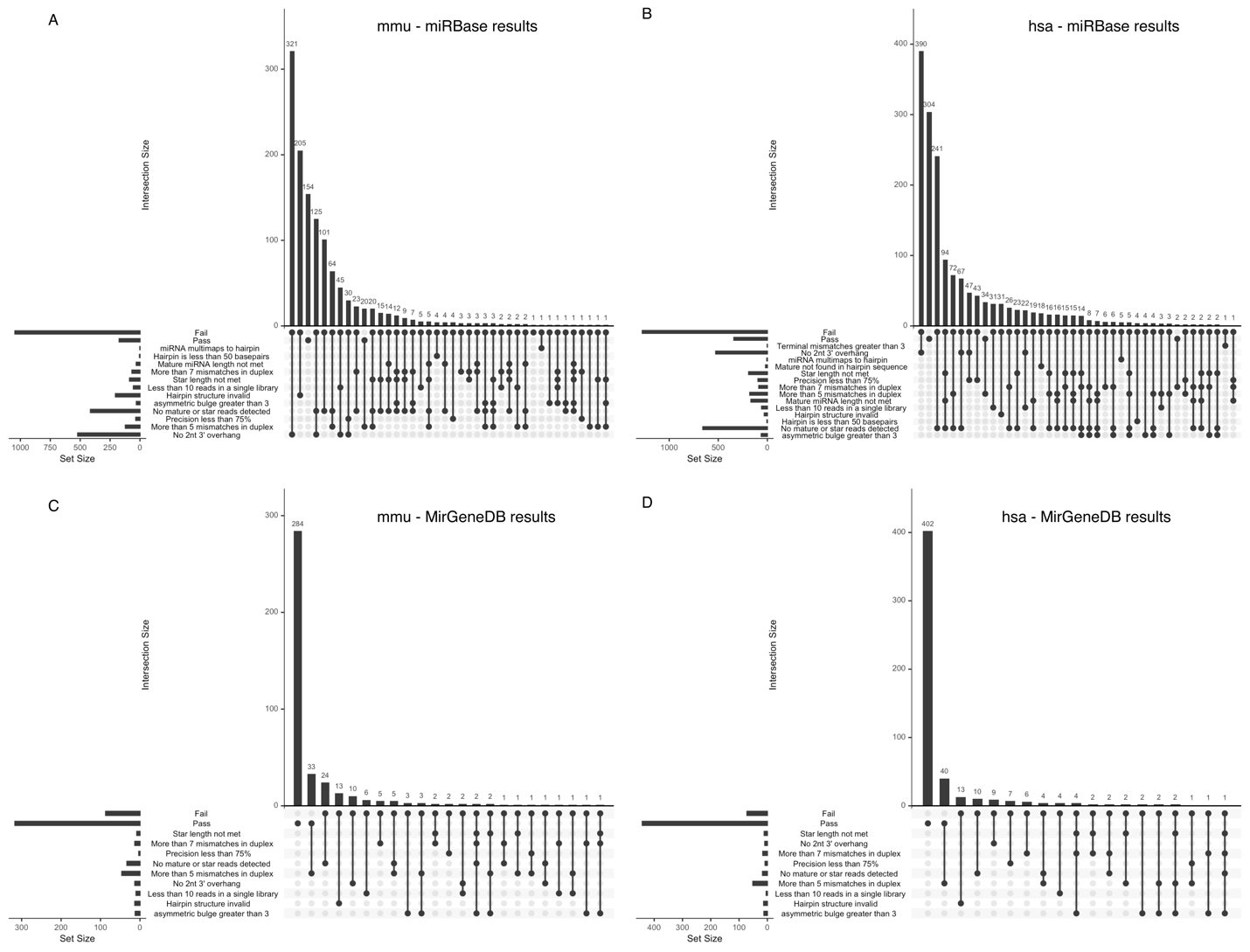


**Supplemental Figure S2.** Upset plot of result and flags for *MIRNAs* sourced from miRBase and MirGeneDB for animal species. See Supplemental File S2 for source data. (A) *Mus musculus* (mmu) mirbase *MIRNA* results and flags. (B) *Homo sapiens* (hsa) mirbase *MIRNA* results and flags. (C) mmu MirGeneDB *MIRNA* results and flags. (D) hsa MirGeneDB *MIRNA* results and flags.
