# Supplementary material for: miRScore: A rapid and precise microRNA validation tool
Source: PLoS Comput Biol. 2025 Nov 3;21(11):e1013663. doi: 10.1371/journal.pcbi.1013663 (PMC12594335; doi:10.1371/journal.pcbi.1013663)
Supplement: S3 Fig — (A) Plot of Hsa-Mir-9-P1 from MirGeneDB with annotated miRNA (orange) and miRNA* (blue). (B) Plot of hsa-let-7a-1 from miRBase with annotated miRNA (orange) and miRNA* (blue). (DOCX) [file pcbi.1013663.s010.docx]

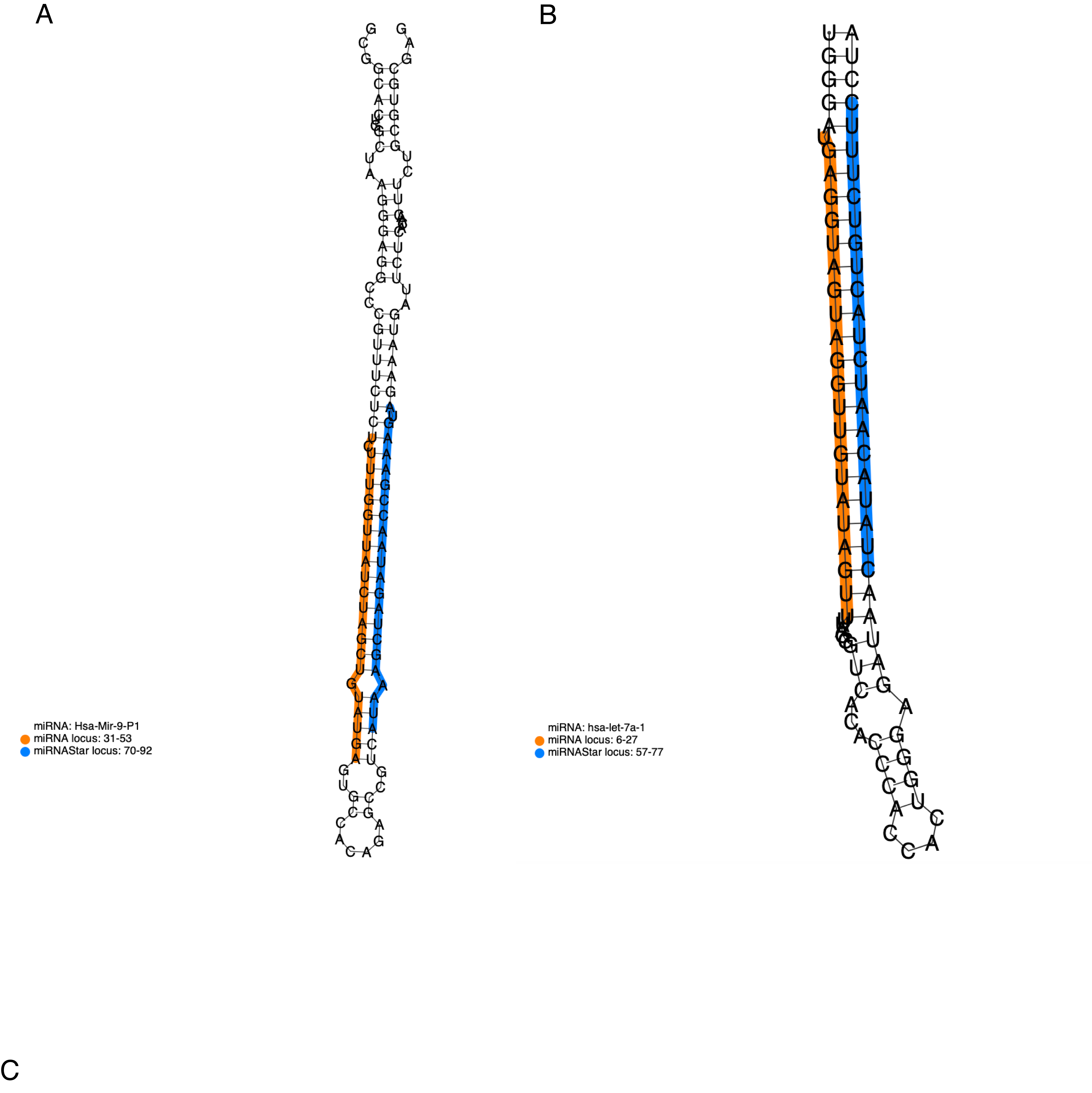
 **Supplemental Figure S3.** RNAplots of *MIRNA* secondary structures. (A) Plot of Hsa-Mir-9-P1 from MirGeneDB with annotated miRNA (orange) and miRNA* (blue). (B) Plot of hsa-let-7a-1 from miRBase with annotated miRNA (orange) and miRNA* (blue).
