# Supplementary material for: miRScore: A rapid and precise microRNA validation tool
Source: PLoS Comput Biol. 2025 Nov 3;21(11):e1013663. doi: 10.1371/journal.pcbi.1013663 (PMC12594335; doi:10.1371/journal.pcbi.1013663)
Supplement: S4 Fig — See S3 File (A) Arabidopsis thaliana (ath) MIRNA results and flags. (B) Oryza sativa (osa) MIRNA results and flags. (C) Zea mays (zma) MIRNA results and flags. (DOCX) [file pcbi.1013663.s011.docx]

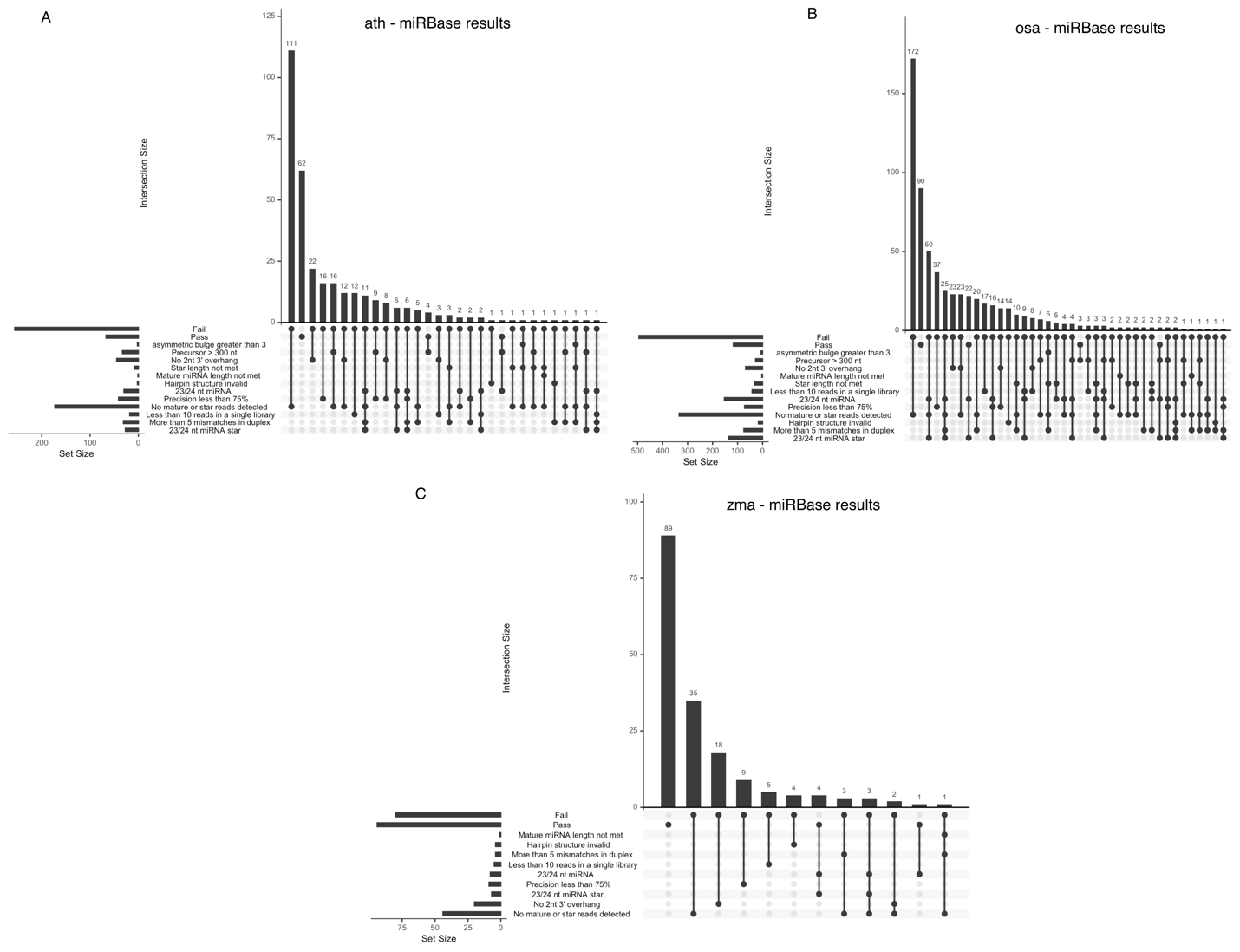


**Supplemental Figure S4.** Upset plot of results and flags for *MIRNAs* sourced from miRBase for plant species. See Supplemental File S4 (A) *Arabidopsis thaliana* (ath) *MIRNA* results and flags. (B) *Oryza sativa* (osa) *MIRNA* results and flags. (C) *Zea mays* (zma) *MIRNA* results and flags.
