# Supplementary material for: miRScore: A rapid and precise microRNA validation tool
Source: PLoS Comput Biol. 2025 Nov 3;21(11):e1013663. doi: 10.1371/journal.pcbi.1013663 (PMC12594335; doi:10.1371/journal.pcbi.1013663)
Supplement: Attachment — Submitted filename: Reviewer_comments.docx [file pcbi.1013663.s013.docx]

*Thank you to the editor(s) and the reviewers for the time spent with our manuscript, and for the suggestions for improvement. We have made several changes to the manuscript and we believe it has been significantly improved. Below please find your original comments (black text) and our responses (red italic text).*

**Editor comments:**

Thank you for submitting your manuscript to PLOS Computational Biology. The manuscript was overall well received by the reviewers and considered to be of wider interest, however they each noted weaknesses in the work and areas in need of clarification. In some parts, a better recognition of the existing literature is required.

*We have edited as suggested to treat the literature more fairly in this area, specifically by citing several of the studies suggested by reviewer 1; details are noted below.*

Reviewers noted that if the results strongly disagree with reference databases, then a higher level of evidence is required.

*We have clarified this point in the revision: A good portion of the failures for existing database entries stem from choice of sRNA-seq expression data. See lines 284-290, 327-337. As for the remainder, it is well established that miRBase in particular has a substantial number of annotations that are in fact not miRNAs. Thus, there is not truly a disagreement in the sense that the reasons for high failure rates are already well-known.*

Moreover the rigour of the work could be improved by adopting enhanced computational reproducibility practices.

*This has been addressed in the revision by addition of detailed commands and versions of all tools used and by inclusion of raw data underlying all plots and code used to make all plots. See Supplemental Files S6-8.*

A more detailed comparison to existing tools/databases and thorough discussion of the discrepancies is required.

*This has been done; see especially the use of a second large database, miRGeneDB, in the revised Figure 2.*

**Reviewers' comments:**

Reviewer's Responses to Questions

**Comments to the Authors:
Please note here if the review is uploaded as an attachment.**

Reviewer #1: Vanek et al (miRScore: a rapid and precise microRNA validation tool) present a computational tool mirScore for assessing the validity of microRNA predictions in plants and animals. Based on 5 selected species they show that the vast majority of annotated microRNAs in miRBase are incorrect (Figure 2) and also test novel candidates in 4 species (Fig 4).

*While a large number of miRNAs annotated on miRBase fail for structural reasons, those that fail for reads and precision are dependent on the specific sRNA-seq data submitted to miRScore. A more comprehensive, large-scale test including many tissues/cell types would likely result in a higher rate of passing. This has been clarified in lines 327-337.*

While the tool seems useful, it is unclear if the authors imply that human has only 92 microRNAs (Table3)?

*We did not intend to imply this. It has been clarified in lines 327-343 that these results (particularly in the case of expression-based failure) are dependent on the sRNA-seq libraries submitted to miRScore*

Regardless, the lack of scholarship in ignoring the literature and efforts by others such as Bracken (e.g. <https://www.biorxiv.org/content/10.1101/2024.12.05.626958v1.abstract>), Corey (e.g. <https://www.biorxiv.org/content/10.1101/2022.10.18.512653v1.abstract>) and our work (discloser: I am Bastian Fromm), especially regarding MirGeneDB & annotation criteria for animals is sobering (e.g. <https://rnajournal.cshlp.org/content/28/6/781.short> & <https://www.annualreviews.org/content/journals/10.1146/annurev-genet-120213-092023>). See also refs below

Thank you for this. We have improved our treatment of the literature including properly citing these works.

Given that the authors SGJ and MJA run their own databases, it is further not understandable why the direct consequences of the proposed tool: the high false-positives in miRBase and possibly the other database, are not highlighted and acted upon by cleaning them up.

*The clean-up effort is outside the scope of this study; here we are simply describing miRScore as an independent validation tool.*

In order to accommodate my critiques, the authors could run their tool on MirGeneDB species. In our latest release (Clarke 2025, NAR), we provide smallRNA seq data.

*Thank you for the suggestion, this has been done. See revised Figure 2.*

1. Castellano, L and Stebbing, J (2013). Deep sequencing of small RNAs identifies canonical and non-canonical miRNA and endogenous siRNAs in mammalian somatic tissues. Nucleic Acids Res. 41: 3339–3351.
2. Chiang, HR, Schoenfeld, LW, Ruby, JG, Auyeung, VC, Spies, N, Baek, D, et al. (2010). Mammalian microRNAs: experimental evaluation of novel and previously annotated genes. Genes Dev. 24: 992–1009.
3. Jones-Rhoades, MW (2012). Conservation and divergence in plant microRNAs. Plant Mol. Biol. 80: 3–16.
4. Ludwig, N, Becker, M, Schumann, T, Speer, T, Fehlmann, T, Keller, A, et al. (2017). Bias in recent miRBase annotations potentially associated with RNA quality issues. Sci. Rep. 7: 5162.
5. Langenberger, D, Bartschat, S, Hertel, J, Hoffmann, S, Tafer, H and Stadler, PF (2011). MicroRNA or Not MicroRNA? Advances in Bioinformatics and Computational Biology, Springer Berlin Heidelberg: pp 1–9.
6. Meng, Y, Shao, C, Wang, H and Chen, M (2012). Are all the miRBase-registered microRNAs true? A structure- and expression-based re-examination in plants. RNA Biol. 9: 249–253.
7. Tarver, JE, Donoghue, PC and Peterson, KJ (2012). Do miRNAs have a deep evolutionary history? Bioessays 34: 857–866.
8. Taylor, RS, Tarver, JE, Hiscock, SJ and Donoghue, PC (2014). Evolutionary history of plant microRNAs. Trends Plant Sci. doi:10.1016/j.tplants.2013.11.008.
9. Wang, X and Liu, XS (2011). Systematic Curation of miRBase Annotation Using Integrated Small RNA High-Throughput Sequencing Data for C. elegans and Drosophila. Front. Genet. 2: 25.
10. Fromm, B, Billipp, T, Peck, LE and Johansen, M (2015). A uniform system for the annotation of vertebrate microRNA genes and the evolution of the human microRNAome. Annual review ofat .
11. Guo, Z, Kuang, Z, Wang, Y, Zhao, Y, Tao, Y, Cheng, C, et al. (2020). PmiREN: a comprehensive encyclopedia of plant miRNAs. Nucleic Acids Res. 48: D1114–D1121.
12. Fromm, B, Domanska, D, Høye, E, Ovchinnikov, V, Kang, W, Aparicio-Puerta, E, et al. (2019). MirGeneDB 2.0: the metazoan microRNA complement. Nucleic Acids Res.: 258749.
13. Fromm, B, Keller, A, Yang, X, Friedlander, MR, Peterson, KJ and Griffiths-Jones, S (2020). Quo vadis microRNAs? Trends Genet. 36: 461–463.
14. Fromm, B, Høye, E, Domanska, D, Zhong, X, Aparicio-Puerta, E, Ovchinnikov, V, et al. (2022). MirGeneDB 2.1: toward a complete sampling of all major animal phyla. Nucleic

*Thank you for the reading list. We have incorporated several of these citations in the revised manuscript.*

Reviewer #2: # Review for Vanek et al 2025

This manuscript describes miRScore, a software allowing quality control of novel or existing miRNA annotations. In that extent, it uses both structural features and sRNA-seq libraries to assess the quality of miRNA annotations. It is demonstrated by applying it on both existing annotations from the miRBase database and novel annotations produced by state of the art software. It is a welcome addition to the miRNA sofware tooltip, given the increasing number of false positive miRNAs submitted to databases. The paper is well written and does a good job of explaining how the software works However, I have two main concerns that I'd like to be at least partially adressed before considering the paper ready for publication

\## Main comments :

\### Comparison to mirBase annotations

The first part of the results demonstrate the pertinence of the software when applied on the miRBase database, which is the generalist reference database for microRNAs.

On this database, for four model species, miRScore dives a "Pass" to between 6 and 54% of registered sequences in miRBase. Annotations failing because of low expression are easily understandable because of the low number of samples (5) used to validate them.

However, the data shows that more than 50% of annotated miRNAs in miRBase for mouse and human don't have a correct miRNA structure (mostly because of missing 3' overhang). Since miRBase is regarded as a reference database

*miRBase is well-known to have a number of false positives, so this is not unexpected. See the citations we make in the manuscript (24,27-31)*

, I think the author need to better support these results, and I have the following remarks :

- Looking at a failing MIRNA like hsa-mir-25, and at the code, I understand that you require a 2nt overhang to have a matching sequence on the hanging part. I thought it was not necessary to see an overhang here but I'm not sure.

*A Drosha/Dicer-processed (animals) or Dicer-Like-processed (plants) miR/miR* duplex with 2nt 3’ overhang is a widely accepted criterion for miRNA biogenesis in animals and plants.*

- While mirBase is very used, they are actually other databases that undewent more curation. In mirGeneDB, I could find at least two genes (hsa-mir-202 and hsa-mir-511) whose mature/star where corrected and show an overhang. I think it would make a lot of sense to run miRScore mirGeneDB, with an opportunity to use miRScore to highlight the difference between miRBase and a curated database.

*Thank you for the suggestion. We have run two species from miRGeneDB to compare to the analysis done in miRBase annotations. Please see revised Figure 2.*

I'm also very pleased to see that hsa-mir-202 has an alternative version in Supp S2 that matches mirGeneDB, nice. Maybe it's something to follow to demonstrate how miRScore can improve an annotation?

*We agree! We’ve added to Figure 2 to highlight this and included more discussion about this feature in lines 215-224.*

\## Reproducibility of the results

While the GitHub repository includes a working minimal example, I think the paper would benefit of making the results of the paper more reproducible. I tried on my side to run miRScore on the full miRNA repertoire of a species in miRBase, and could not easily do it (see log below), maybe because I missed some preprocessing step? Could you please include in the documentation a complete example of how to run miRscore on a mirBase species just as in the paper?

*We did not design miRScore to only examine records from miRBase or miRGeneDB, but we certainly acknowledge that this may be a common use case. Both databases have inconsistencies and idiosyncrasies in their microRNA and hairpin naming schemes that prevent input of "raw" files into miRScore. In this revision we’ve included a markdown file in supplementals to explain how to prepare miRBase and miRGeneDB data for running miRScore (Supplemental File S7) and this information has also been added to the README on github.*

```
---
miRScore version 0.3.2
Options:
'Mature file' ath.mature.fa
'Hairpin file' ath.hairpin.fa
'Threads' 1
'Kingdom' plant
'Fastqs' ['fastq/SRR218085.fastq.gz', 'fastq/SRR218092.fastq.gz', 'fastq/SRR218096.fastq.gz', 'fastq/SRR218098.fastq.gz', 'fastq/SRR218099.fastq.gz']
'Output directory' miRScore_output/

---

miRNAs submitted: 326

Checking hairpin and miRNA sequences...

Traceback (most recent call last):
File "/home/cguyomar/conda/env_mirscore/bin/miRScore", line 1557, in 
main()
File "/home/cguyomar/conda/env_mirscore/bin/miRScore", line 913, in main
star=hairpin[(starpos[0]-1):starpos[1]].upper()
~~~~~~~~~~^~
TypeError: unsupported operand type(s) for -: 'NoneType' and 'int'
```

Also, for the sake of reproducibility, it would be nice to deposit somewhere :

- The outputs and commands of ShortStack/miRador/mirDeep-P2

*A markdown file of these analyses is now provided as Supplemental File S6*

- The scripts to generate the figures of the paper

*Provided in Supplemental File S8.*

\# Minor comments

l168 : Could you please give the options used for Bowtie (if different from default)

*This is now in the methods. These options were available in the README as well. By default, bowtie runs as follows:*

*bowtie -v 0 -a –no-unal –norc -x hairpin -S*

l172 : You are using a raw 10 read expression threshold. Shouldn't you perform a minimal amount of normalization like RPM to account for library size differences?

*We considered this at length during development and ultimately decided that the most inclusive approach to capture lowly expressed miRNAs would be to use raw reads and a read floor. The use of normalized reads is unnecessary in this instance as we do not compare between libraries (i.e. we are not calling differential expression), but on a per-library basis.*

l173 : It is actually common that the passenger strand is not detected at all. While I understand the concern, I find that "No star reads detected" could deserve its own flag, less worrying as "No mature or star reads detected".

*We also considered this at length during development. However, it added an additional flag to consider for subsequent data curation and analysis. Since the criteria requires both miR/miR* reads present regardless, we felt it was more effective to use a single flag. Note that the results table also clearly lists number of miRNA and miRNA* reads.*

l174 : Could precision be impacted by isomirs? Is an exact matching required to consider a read as "miRNA duplex read"?

*Yes isomirs can impact precision. We account for some variation in the reference mature sequence when it comes to counting reads. This is what we refer to as ‘variance’ within the text and README (Supplemental Figure S1). We include 1-nt positional variance on either side of the reported miRNA sequence. Isomirs with greater than ~1 nt variance will not be easily passed by miRScore. The difficulty in miRScore passing non-canonical miRNAs, such as isomiRs, is now mentioned at lines 286-292.*

l236 : I'm not sure to understand why is reference 38 here. I understand it is about the evolutionary dynamics of microRNAs and their targets and not their structural features?

*Fair point; this reference has been removed.*

I think what could be mentionned here is non canonical miRNAs pathway like mirtrons. Could they be a part of the explanation for the many missing 3' overhangs?

*We agree; this is now mentioned briefly at line 286-292.*

l275 : Since ShortStack has been developped by the last author, could I be that it already includes structural constraints analog to those of miRScore when predicting MIRNAs?

*This has been acknowledged in the text and expanded on lines 376-388.*

Reviewer #3:

1) The theme of the manuscript 'miRScore: a rapid and precise microRNA validation tool' is relevant to the Journal.
2) The basis of Criteria for endogenous miRNAs in plants and animals as mentioned in Table 1 needs clarification. Demonstration with specific known miRNA in different species can be conducted to illustrate stringency of the criteria. Though similar attempt with group of miRNA has been conducted as mentioned in Table 3.

*We’ve updated our discussions (lines 254-292, 327-343) on specific miRNAs, included a supplemental to demonstrate read criteria (Supplements Figure S1). Figure 3 also demonstrates criteria.*

3) Performance of validation of miRDeep-2 versus Shortstack merits discussion.

*We have expanded on the different validation rates by miRScore (lines 362-373) and included the code used for running these annotations (Supplemental File S6). A detailed investigation into performance metrics of de novo software such as ShortStack and miRDeep-2 is beyond the scope of this study.*

4) A comparative table of performance of miRScore with entries from difference databases will be useful.

*This has been included with animal miRNAs from MirGeneDB; see revised Figure 2. Thank you for the suggestion.*

**Have the authors made all data and (if applicable) computational code underlying the findings in their manuscript fully available?**
The [PLOS Data policy](https://nam10.safelinks.protection.outlook.com/?url=https%3A%2F%2Ftrack.editorialmanager.com%2FCL0%2Fhttps%3A%252F%252Fjournals.plos.org%252Fploscompbiol%252Fs%252Fmaterials-and-software-sharing%2F1%2F010f0195f70b1edc-8d537463-06c6-42e6-8544-ef240c4a8be4-000000%2FKLGyx3GGpGRpVIAYmzGGE3HdSjGtJB9YkG1JUUtnOZs%3D205&data=05%7C02%7Caez35%40psu.edu%7C92762bdb9e3d48d249c308dd71f81a83%7C7cf48d453ddb4389a9c1c115526eb52e%7C0%7C0%7C638792032637522147%7CUnknown%7CTWFpbGZsb3d8eyJFbXB0eU1hcGkiOnRydWUsIlYiOiIwLjAuMDAwMCIsIlAiOiJXaW4zMiIsIkFOIjoiTWFpbCIsIldUIjoyfQ%3D%3D%7C0%7C%7C%7C&sdata=9hyj51X2lyt9dRJUBIznJyy3iaOAGDSrZZYfpLKE34I%3D&reserved=0) requires authors to make all data and code underlying the findings described in their manuscript fully available without restriction, with rare exception (please refer to the Data Availability Statement in the manuscript PDF file). The data and code should be provided as part of the manuscript or its supporting information, or deposited to a public repository. For example, in addition to summary statistics, the data points behind means, medians and variance measures should be available. If there are restrictions on publicly sharing data or code —e.g. participant privacy or use of data from a third party—those must be specified.

Reviewer #1: Yes

Reviewer #2: **No:** The code to reproduce the paper results is missing

*This has been addressed; See Supplement File S6-8*

Reviewer #3: Yes

PLOS authors have the option to publish the peer review history of their article ([what does this mean?](https://nam10.safelinks.protection.outlook.com/?url=https%3A%2F%2Ftrack.editorialmanager.com%2FCL0%2Fhttps%3A%252F%252Fjournals.plos.org%252Fploscompbiol%252Fs%252Feditorial-and-peer-review-process%2523loc-peer-review-history%2F1%2F010f0195f70b1edc-8d537463-06c6-42e6-8544-ef240c4a8be4-000000%2FBCIfCYnMQfXfjN7sC8ABBmn7-3V0KsJs02JuZSr5IT0%3D205&data=05%7C02%7Caez35%40psu.edu%7C92762bdb9e3d48d249c308dd71f81a83%7C7cf48d453ddb4389a9c1c115526eb52e%7C0%7C0%7C638792032637543116%7CUnknown%7CTWFpbGZsb3d8eyJFbXB0eU1hcGkiOnRydWUsIlYiOiIwLjAuMDAwMCIsIlAiOiJXaW4zMiIsIkFOIjoiTWFpbCIsIldUIjoyfQ%3D%3D%7C0%7C%7C%7C&sdata=Z91aiHxOS%2BZT0gwUUOvDlmxpD379QS1BMZP%2FzvO4Wy0%3D&reserved=0)). If published, this will include your full peer review and any attached files.


If you choose “no”, your identity will remain anonymous but your review may still be made public.


**Do you want your identity to be public for this peer review?** For information about this choice, including consent withdrawal, please see our [Privacy Policy](https://nam10.safelinks.protection.outlook.com/?url=https%3A%2F%2Ftrack.editorialmanager.com%2FCL0%2Fhttps%3A%252F%252Fwww.plos.org%252Fprivacy-policy%2F1%2F010f0195f70b1edc-8d537463-06c6-42e6-8544-ef240c4a8be4-000000%2FN8voX2nNOx_g1gmPl57cDkA968n1v2IZiVic1Tc5dZU%3D205&data=05%7C02%7Caez35%40psu.edu%7C92762bdb9e3d48d249c308dd71f81a83%7C7cf48d453ddb4389a9c1c115526eb52e%7C0%7C0%7C638792032637559196%7CUnknown%7CTWFpbGZsb3d8eyJFbXB0eU1hcGkiOnRydWUsIlYiOiIwLjAuMDAwMCIsIlAiOiJXaW4zMiIsIkFOIjoiTWFpbCIsIldUIjoyfQ%3D%3D%7C0%7C%7C%7C&sdata=rQUYq0P93GvGbDbAgPpMtmltMCDNiWw3awf3uAM5efU%3D&reserved=0).

Reviewer #1: **Yes:** Bastian Fromm

Reviewer #2: No

Reviewer #3: **Yes:** Ashwin Kotnis
