# Supplementary material for: miRScore: A rapid and precise microRNA validation tool
Source: PLoS Comput Biol. 2025 Nov 3;21(11):e1013663. doi: 10.1371/journal.pcbi.1013663 (PMC12594335; doi:10.1371/journal.pcbi.1013663)
Supplement: Attachment — Submitted filename: ReviewersComments_2.docx [file pcbi.1013663.s014.docx]

Thank you again to the Editors and Reviewers for their time and constructive comments. We have revised the manuscript to better address the remaining concerns. Below, the original comments are in black font, and our responses are in red font. Text in *red italics* indicates direct quotes from the revised manuscript.

**Reviewers' comments:**

Reviewer's Responses to Questions

**Comments to the Authors:
Please note here if the review is uploaded as an attachment.**

Reviewer #1: I am happy to see the authors have added two species from MirGeneDB as a comparison, but I am sceptical whether the functionality of the tool "a tool to quickly analyze novel and annotated MIRNAs for further analysis or submission to a database" has sufficiently been shown, or, for this matter, is actually applied by the authors themselves.

The listed annotation criteria (Table 1) seem arbitrary and - for animals - somewhat out of touch with studies by Bartel, Kim and also us on annotation criteria for animals. For example did we show that mature microRNAs in animals can be between 20 and 27 nucleotides in length (Fromm 2015 AnnRev)

We allow 20-24 nucleotides in plants because that is the known size range of Dicer-Like produced RNAs through both genetic and biochemical analyses. The Fromm et al. (2015) review actually states a size range of 20-27 (see in that review Box 1 "CONSISTENT SET OF CRITERIA FOR THE ANNOTATION OF METAZOAN miRNAs".), which is the range the miRScore also uses for animals.

and that there is no "hard" limit on microRNA hairpin lengths in animals (Fromm 2013, MBE and e.g. see also https://mirgenedb.org/show/dpu/Mir-375.

Either way: where does 200nt and 300nt for max length come from?

miRScore does not use a hard limit on hairpin length. When miRScore encounters hairpin lengths longer than 200 (animals) or 300 nt (plants), it raises a warning but will not fail the locus solely for having a long hairpin. This is stated in the README file and there are examples of passed loci with long hairpin warnings in the supplemental files. We have revised the manuscript to make this clearer, most prominently by adding a new table (Table 2 in this revised manuscript) clearly describing the flags that miRScore produces, and their consequences (either "fail" or "warning"). A better description of flags producing "fail" vs. "warning" is also included in the revised text (*miRScore utilizes a “pass/fail” system of reporting: each input MIRNA locus will ultimately either pass or fail. In some cases, one or more warnings may be raised for a "passed" entry if certain atypical features are present. A full list of flags and their explanations can be found in Table 2 and in the miRScore README. If one or more flags with a "fail" result are present, the locus will fail. Loci with no flags will pass, as will loci that have one or more flags associated with a "warning" result but no flags with a "fail" result (Table 2).*).

We agree there is no hard limit on miRNA hairpin length, but very long hairpins are quite unusual, and long sequences reduce the confidence in RNA secondary structure prediction. Spurious long hairpins are a frequent source of bad annotations from several genome-wide miRNA annotation tools. For these reasons, we feel a warning is appropriate for such loci.

Where the other criteria?

The other criteria we use are our best synthesis of the literature cited, as we state in the manuscript: *The validation process is based on a set of previously described criteria which can be categorized as either structural (based on the predicted RNA secondary structure of the precursor) or expression (based on observations of miRNA and miRNA* abundance) (Table 1)(23–25,27,38,39)*

For the plant microRNA part, I am aware that there are at least 3 competing microRNA databases for plants (including miRBase), but it seems the annotations are not compared to other databases, why?

I am curious why are you only looking at so few species, and why at two very closely related species for animals? Why canæt you do all miRBase and plant DB species?

The purpose of this study is not to comprehensively examine and pass judgment on every microRNA that has ever been annotated, or to look at all possible databases. That is beyond the scope of this study for several reasons:

- miRScore depends on the input sRNA-seq data. A locus that fails in one analysis with one set of sRNA-seq data may pass in a second analysis, if the second analysis uses sRNA-seq data where the miRNA and miRNA* are more abundant. A truly comprehensive analysis of every annotation would require analysis of many thousands of sRNA-seq libraries for every single species (hundreds or more species).
- In this study, we used selected annotations from existing databases as input as test cases to evaluate miRScore performance. These selected annotations were also each manually examined (by hand), a process that is extremely time intensive. The comparison between miRScore's classification and the manual classification is what underlies the performance analysis in Table 4. It is not realistic to extend this type of performance analysis to hundreds of species and tens of thousands (or more) loci.
- Most importantly: The purpose here is not to retroactively pass judgement on prior annotations; the purpose instead is to test the performance of the tool and document the performance for the community. Because this manuscript is a "Software" manuscript, we contend that this is an appropriate scope.

Finally, to come back to the scope of the tool to be informing on existing databases' annotations: the authors (SGJ, BCM, MJA) host leading microRNA / sncRNA repositories that are only partially tested here and they found for miRBase massive numbers of false-positives (as many others did before) - Why are the authors not acting on their results and remove those false entries from their DBs?

We understand the focus on existing annotations because we have used some of them as test cases for miRScore performance. We emphasize however that our main purpose for creating miRScore is to act as a filter for NEW, incoming submissions to microRNA databases. We have made numerous changes to the text throughout this revision to better emphasize this point. For instance, the first paragraph of the Results and Discussion now reads "*miRScore is primarily designed as a quick secondary filter to analyze new MIRNA annotations prior to submission or acceptance into a permanent repository. However, existing repositories contain multitudes of diverse annotations that have already been peer-reviewed and curated and as such are a good source of input data to evaluate miRScore performance. Therefore, for initial performance testing we obtained MIRNA annotations from miRBase version 22.1 and MirGeneDB version 3.0 from two animal species (Homo sapiens and Mus musculus) and three plant species from miRBase version 22.1 (Arabidopsis thaliana, Oryza sativa, and Zea mays) (Table 3)*". Behind the scenes, we are considering whether to implement miRScore as an automatic screen for new submissions to miRBase. That's not mentioned in this manuscript because that design process is ongoing. In this revision we've changed several parts of the text to make the purpose clearer.

We are not removing entries from miRBase right now because a miRScore "fail" during our performance analysis is not on its own enough evidence to warrant removal of miRNA entries that, after all, passed peer review and curation sometime in the past. This would be a more involved process, including deep searching of the literature (has this microRNA been functionally characterized? for instance), and the scouring of ALL possible small RNA-seq sources (because a miRScore "pass" requires ample accumulation of miRNA/miRNA* in sRNA-seq data). It probably would require consulting experts on individual species or kingdoms before fully removing entries; none of the authors can confidently claim to be experts on all of microRNA variations seen in every species.

We are certainly sympathetic to this desire; we agree that this is a worthy goal. However, a software manuscript, for software designed primarily as a filter for new incoming annotations, is not the venue for a comprehensive housecleaning of entries in the vast array of existing miRNA annotations.

The inclusion of false-positives has had a really negative influence on the field and miRScore is the perfect opportunity to rectify this.

We agree that bad miRNA annotations are an issue, and we are happy that you think miRScore might help.

MINOR: please spell MirGeneDB consistently MirGeneDB.

Fixed.

Reviewer #2: I am satisfied with the revisions made by the authors, which have significantly improved the quality of the paper. I have no further comments at this time.

Thank you for your time and consideration.
