# Supplementary material for: miRScore: A rapid and precise microRNA validation tool
Source: PLoS Comput Biol. 2025 Nov 3;21(11):e1013663. doi: 10.1371/journal.pcbi.1013663 (PMC12594335; doi:10.1371/journal.pcbi.1013663)
Supplement: Attachment — Submitted filename: miRScore_Revision3_Rebuttal.docx [file pcbi.1013663.s016.docx]

>>> Thank you again for the time spent with our manuscript and for the opportunity to submit a revised version. Below, find your original comments in black, and our responses in red font.

**Additional Editor Comments :**

As this article has been in the review process for a long time, I am keen to provide straightforward direction to progress it hopefully to acceptance quickly. As the reviewer states, the rescue feature of miRscore appears to be identifying some problematic loci as true miRNA genes. In fact a large fraction (~50%) of recently rejected miRbase microRNAs appear to be true miRNAs using miRscore. As the reviewer states, this could cause confusion in the field. Therefore for the benefit of the field, miRscore criteria should be adjusted to exclude the known problematic loci including the ones named by the reviewer.

>>> We have made a new release of miRScore in which the "rescue" feature in question is no longer run by default. (See release 0.3.4 on the github at https://github.com/Aez35/miRScore). Instead, users will have to opt in, and the updated README has warnings to treat any "rescued" loci with due diligence. We have made corresponding changes in the manuscript: Removal of the "rescue" figures (Former panels 2C and 2D) and supplemental data and a re-configured and much shorter discussion of this now optional feature. The shorter discussion is confined to the methods section and includes a warning to users to check any rescued loci carefully before making a final determination.

As hard thresholds are rarely perfect classifiers, authors could consider tiered classification (eg: high confidence, low confidence and no-miR loci) where the doubtful miRs could be placed in the low confidence group.

>>> miRScore doesn't exactly use these terms, but it does already have something similar in spirit: "Warning" flags are issued for loci that do pass, but have unusual features. See Table 2 for details.

Authors could also include a passage in the introduction about the clean-up of miRbase to remove some false/problematic miRs.

>>> Done.

Note: If the reviewer comments include a recommendation to cite specific previously published works, please review and evaluate these publications to determine whether they are relevant and should be cited. There is no requirement to cite these works unless the editor has indicated otherwise.


**Reviewers' comments:**

Reviewer's Responses to Questions

**Comments to the Authors:
Please note that one review is uploaded as an attachment.**

Reviewer #1: I was reluctant to follow the argument about applying miRscore to the whole of miRBase or other plant databases, but could see (from experience) that this would be a lot of work and, hence, was to be OK with the revisions. However, then I noticed the "rescued" miRBase microRNAs and had a brief look at the entries rescued which really was surprising and a bit shocking to me:

Not only were there many rescued "microRNAs" previously rejected by us , showing that miRscore actually could be detrimental to previous efforts to clean up the number of misannotated human loci, but in addition (highlighted in the figure I uploaded) were there clear cases of very bad examples, such as "mir-548" (a transposon with many hits), "mir-1258" with more than hundred hits in the genome, mir-3130 with a palindromic (very unusual!) sequence and mir-6132 with mature reads in mirbase below 20nts....

I think the rescuing feature is counterintuitive and will open floodgates of hundreds of novel "miRNAs" to be validated with miRscore - something that shoulg be avoided for the sake of the field!

>>> We have made a new release of miRScore in which the "rescue" feature in question is no longer run by default. (See release 0.3.4 on the github at https://github.com/Aez35/miRScore). Instead, users will have to opt in, and the updated README has warnings to treat any "rescued" loci with due diligence. We have made corresponding changes in the manuscript: Removal of the "rescue" figures (Former panels 2C and 2D) and supplemental data and a re-configured and much shorter discussion of this now optional feature. The shorter discussion is confined to the methods section and includes a warning to users to check any rescued loci carefully before making a final determination.
