# Supplementary material for: miRScore: A rapid and precise microRNA validation tool
Source: PLoS Comput Biol. 2025 Nov 3;21(11):e1013663. doi: 10.1371/journal.pcbi.1013663 (PMC12594335; doi:10.1371/journal.pcbi.1013663)
Supplement: Attachment — Submitted filename: miRScore_Revision4_Rebuttal.docx [file pcbi.1013663.s017.docx]

>>> Thank you again for the time spent with our manuscript and for the opportunity to submit a revised version. Below, find your original comments in black, and our responses in red font.

**IMPORTANT NOTE:** The "track changes" document for this revision has almost no tracked changes because the edits were so minimal in this revision .. just deleted some stray punctuation in the Figure 2 caption. The main change is the removal of the two panels on Figure 2 per the reviewer's comment.

Reviewer #1: I appreciate that the authors took my criticism seriously. We want to improve the standards in the field, not make it worse. To this end the authors should remove Figure 2C and D.

>>> Thank you, you are correct, these two panels should have been removed in the previous revision. They have been removed now.
